# Multiple Functions of Fubp1 in Cell Cycle Progression and Cell Survival

**DOI:** 10.3390/cells9061347

**Published:** 2020-05-28

**Authors:** Mingyu Kang, Hyeon Ji Kim, Tae-Jun Kim, Jin-Seok Byun, Jae-Ho Lee, Deok Heon Lee, Wanil Kim, Do-Yeon Kim

**Affiliations:** 1 Department of Pharmacology, School of Dentistry, Kyungpook National University, Daegu 700-412, Korea; alsrb5788@naver.com (M.K.); guswl1634@naver.com (H.J.K.); toy5988@naver.com (T.-J.K.); 2 Department of Oral Medicine, School of Dentistry, Kyungpook National University, Daegu 700-412, Korea; jsbyun@knu.ac.kr; 3 Department of Anatomy, Keimyung University School of Medicine, Daegu 42601, Korea; anato82@dsmc.or.kr; 4 Department of Thoracic and Cardiovascular Surgery, Kyungpook National University Hospital, Kyungpook National University School of Medicine, Daegu 42601, Korea; ldhms@naver.com; 5 Department of Cosmetic Science and Technology, College of Bio-industry, Daegu Haany University, Gyeongsan 712-715, Korea; 6 Department of Pharmacology, School of Dentistry, Brain Science and Engineering Institute, Kyungpook National University, Daegu 700-412, Korea

**Keywords:** Fubp1, cell cycle, cyclin A, cell death, lung cancer, double-agent

## Abstract

The discovery of novel and critical genes implicated in malignant development is a topic of high interest in cancer research. Intriguingly, a group of genes named “double-agent” genes were reported to have both oncogenic and tumor-suppressive functions. To date, less than 100 “double-agent” genes have been documented. Fubp1 is a master transcriptional regulator of a subset of genes by interacting with a far upstream element (FUSE). Mounting evidence has collectively demonstrated both the oncogenic and tumor suppressive roles of Fubp1 and the debate regarding its roles in tumorigenesis has been around for several years. Therefore, the detailed molecular mechanisms of Fubp1 need to be determined in each context. In the present study, we showed that the Fubp1 protein level was enriched in the S phase and we identified that Fubp1 deficiency altered cell cycle progression, especially in the S phase, by downregulating the mRNA expression levels of *Ccna* genes encoding cyclin A. Although this Fubp1-cyclin A axis appears to exist in several types of tumors, Fubp1 showed heterogeneous expression patterns among various cancer tissues, suggesting it exhibits multiple and complicated functions in cancer development. In addition, we showed that Fubp1 deficiency confers survival advantages to cells against metabolic stress and anti-cancer drugs, suggesting that Fubp1 may play both positive and negative roles in malignant development.

## 1. Introduction

Fubp1 is a DNA and RNA binding protein that mainly functions in the transcription of its target genes [1]. Although three nuclear localization signals (NLS) confers the enrichment of Fubp1 in the nucleus [2], Fubp1 could translocate to the cytoplasm under various stimuli including viral infection and apoptosis to control mRNA stability or translation of its target genes [3,4]. Increasing evidence has demonstrated the oncogenic role of Fubp1 and deregulated expression of *FUBP1* has been reported in several types of tumors, including hepatocellular carcinoma [5,6], nasopharyngeal carcinoma [7], gastric cancer [8], leukemia [9] and neuroblastoma [10]. The molecular mechanisms by which FUBP1 contributes to tumor propagation are currently being investigated. Among them, the oncogene *MYC* is a well-known downstream target of FUBP1 and abnormal *MYC* overexpression mediated by FUBP1 has been consistently reported by several independent studies in various tumor types [8,11]. However, other studies have reported that the FUBP1-*MYC* axis might not be ubiquitous since *MYC* expression is not altered by FUBP1 silencing in different cell types, such as normal fibroblasts [12], prostate and bladder cancer [13]. Given that a tumor is basically caused by uncontrolled cell cycle progression, it is not surprising that the cell cycle inhibitor *p21* is another main target gene repressed by FUBP1 [6]. However, because *p21* expression was upregulated, rather than downregulated, by FUBP1 in certain circumstances [14], the FUBP1-p21 axis also needs to be further verified. In hematopoietic lineages, FUBP1 cooperates with RUNX1 to facilitate the transcription of *c-KIT* [15]. In short, downstream target selection by Fubp1 seems to occur in a context-dependent manner.

Whether Fubp1 is an oncogene remains controversial. Interestingly, inactivating mutations of *FUBP1* were identified in a substantial fraction of oligodendrogliomas, suggesting the tumor-suppressive role of Fubp1 [16]. In addition, loss-of-function mutations of Fubp1 might contribute to gliomagenesis mediated by lysine-specific demethylase 1 (LSD1)+8a deficiency [17]. Molenaar et al. also described the tumor suppressive effects of *FUBP1* by showing that higher *FUBP1* expression correlated with better survival in all stages of human neuroblastoma [18]. Taken together, Fubp1 likely functions as both a tumor suppressor and an oncogene and the detailed molecular mechanisms of Fubp1 in each context need to be determined.

Dynamic cooperation between cyclins and cyclin-dependent kinases (Cdks) is essential for normal cell cycle progression. Eukaryotic cells have multiple cyclins and each cyclin is associated with a particular phase of the cell cycle. Given the importance of cyclins in cell cycle transitions, both cyclin accumulation and degradation are tightly controlled. For example, cyclin A and cyclin F mRNA levels remain low during G1 but they begin to accumulate at the onset of the S phase. After reaching a peak in G2, the levels of cyclin A and cyclin F decline around mitosis [19,20]. In contrast, the synthesis of cyclin E mRNA is initiated during G1 and then cyclin E is downregulated in the S phase [21].

Because cyclins are critical elements of cell cycle regulation and the disruption of cell cycle control is the main signature of cancer cells, mutation or overexpression of cyclins was frequently observed in a variety of neoplastic diseases. For example, approximately 15% of primary breast cancers accompany the amplification/rearrangement of cyclin D1 [22,23]. Furthermore, over 25% of breast cancers contain cyclin A gene amplification and excessive expression of cyclin A is linked to poor prognosis in breast cancer patients [24]. Notably, increasing evidence has shown that the upregulation of transcripts or proteins is not necessarily caused by chromosome amplification [25,26]. Indeed, while cyclin A gene amplification is found in about a quarter of breast cancers, cyclin A overexpression is observed in over 80% of breast tumor samples [24]. This finding suggest that the dysregulated and excessive expression of cyclins without gene amplification may also be a key factor contributing to tumorigenesis.

Although the function of each cyclin has been well documented, the precise mechanisms regulating the expressions of cyclins are not fully understood. Given that Fubp1 is implicated in tumor development, we validated the role of Fubp1 in cell cycling in this study. We found that Fubp1 protein is more abundant in the S phase and Fubp1 deficiency slowed down cell cycle progression, especially in the S phase, by lowering cyclin A expression. However, we also showed an anti-cancer function of Fubp1 in certain circumstances. Tumor cells are often under the metabolic stress mainly due to their active growth followed by limited nutrient supply. Tumors could also encounter genotoxic stress, including oxidative stress, by antineoplastic drugs. Our data showed that Fubp1 deficiency provides cells with survival advantages against metabolic stress and anti-cancer drug, suggesting that Fubp1 has multiple functions in malignant development.

## 2. Materials and Methods

### 2.1. Plasmid Construction

The lentiCRISPRv2 plasmid was digested and dephosphorylated with BsmBI, followed by gel purification. The lentiCRISPRv2 was a gift from Feng Zhang (Addgene plasmid #52961; http://n2t.net/addgene:52961; RRID: Addgene_52961) [27]. In order to clone the Fubp1 target sequence into the lentiCRISPRv2, a pair of oligos was phosphorylated with T4 Polynucleotide Kinase (NEB, cat no. M0201S) and annealed in thermocycler (incubation in 95 °C for 5 min followed by ramping down to 25 °C at 5 °C/min). Annealed oligos were inserted into digested lentiCRISPRv2 plasmid with T4 DNA ligase (NEB, cat no. M0202S) to generate LC-Fubp1 plasmid. The sequences of the oligos are as follows: 5’-CACCGCCAGCCGAGCCAGACGACGG-3’ and 5’-AAACCCGTCGTCTGGCTCGGCTGGC-3’.

### 2.2. Cell Culture and Generation of Fubp1-Deficient LLC Cells

The NIH3T3 fibroblasts and Lewis lung carcinoma (LLC) cells were maintained in Dulbecco’s modified Eagle’s medium (DMEM) supplemented with 10% fetal bovine serum and 1% penicillin–streptomycin in a humidified atmosphere containing 5% CO_2_ at 37 °C. To generate Fubp1-deficient LLC cells, LC-Fubp1 plasmid was transfected into LLC cells by using lipofectamine 3000 (Thermo Fisher Scientific, Waltham, MA, USA), according to the manufacturer’s instructions. To generate control cells, LC-GFP plasmid was introduced to LLC cells, instead of LC-Fubp1 plasmid. At 2 days after transfection, we started selection with 2 μg/mL puromycin. Puromycin-resistant control and Fubp1-deficient- NIH3T3 and LLC cells were maintained in DMEM supplemented with 10% fetal bovine serum, 1% penicillin–streptomycin and 2 μg/mL puromycin.

### 2.3. Protein Preparation and Immunoblot Analysis

Cells were disrupted directly with laemmli buffer (60 mM Tris-HCl (pH 6.8), 2% (*w*/*v*) sodium dodecyl sulfate (SDS), 10% (*v*/*v*) glycerol and 0.02% (*w*/*v*) bromophenol blue), followed by sonication and heat-denaturation. Cell lysates were subjected to sodium dodecyl sulfate-polyacrylamide gel electrophoresis and transferred to a polyvinylidene fluoride membrane. After blocking the membranes with 5% non-fat dried milk in Tris-buffered saline with Tween^TM^ 20 (TBST) (10 mM Tris, pH 8.0, 150 mM NaCl and 0.5% Tween 20) for 30 min, membranes were washed three times (10 min each) with TBST and incubated with antibodies against Fubp1 (1:1000, Abcam, Cambridge, UK, cat no. ab181111), GAPDH (1:1000, Cusabio, Houston, TX, USA, cat no. CSB-MA000184), phospho-Histone 3 at Ser 10 (1:1000, Cell Signaling, Leiden, The Netherlands, cat no. 3377), cyclin A (1:500, Santacruz, Paso Robles, CA, USA, cat no. sc-596), β actin (1:5000, Sigma Aldrich, St. Louis, MO, USA, cat no. A5316), cyclin B1 (1:500, Santacruz, cat no. sc-245) and cyclin D1 (1:500, Santacruz, cat no. sc-450) overnight at 4 °C. The next day, membranes were washed three times (10 min each) with TBST and incubated with horseradish peroxidase-conjugated anti-mouse (1:10,000, Bethyl Laboratories, Montgomery, TX, USA) or anti-rabbit secondary antibodies (1:5000, Bethyl Laboratories) for 1 h. Membranes were washed three times (10 min each) with TBST and signals were detected with a D-Plus^TM^ ECL Femto system (Dongin LS). The quantification of Western blots was performed with ImageJ. Protein band intensities were first normalized to the loading control band intensities corresponding to the specific protein and further normalized to the first experimental replicate in control (lane 1) samples within each technical replicate (*n* = 3). Biological relevance of the results were confirmed with at least three independent experiments with similar results.

### 2.4. Immunofluorescence

For phospho-Histone 3 immunostaining, cells were fixed with 4% paraformaldehyde and permeabilized with 0.2 % Triton X-100/PBS for 15 min each at room temperature. Cells were washed with cold PBS several times after fixation and permeabilization. After blocking samples with 2% BSA/PBS for 30 min, cells were subjected to immunofluorescence staining with anti-phospho-Histone 3 at Ser 10 (1:200, Cell Signaling) primary antibodies overnight at 4 °C. The next day, cells were washed with cold PBS and incubated with Flamma^®^488-conjugated goat anti-rabbit IgG (Bioacts) for 30 min at room temperature. For nuclear staining, cells were incubated with Hoechst 33342 dye (Thermo Fisher Scientific) for 5 min. Fluorescence signals were visualized with an EVOS FL Auto Imaging System (Thermo Fisher Scientific).

For bromodeoxyuridine (BrdU) staining, cells were labeled with BrdU by incubation with 10 μM of BrdU at 37 °C for 30 min. After washing cells twice with phosphate-buffered saline (PBS), fixation and permeabilization were performed as described above. Cells were treated with 2M HCl for 10 min and then neutralized with 0.1 M sodium borate buffer (pH 8.5) for 30 min at room temperature. After washing and blocking, cells were subjected to immunofluorescence staining with anti-BrdU (1:200, Thermo Fisher Scientific, cat no. MA3-071) primary antibodies at room temperature for 1 h. Cells were washed with cold PBS and incubated with Flamma^®^488-conjugated goat anti-mouse IgG (Bioacts) for 30 min at room temperature. Nuclear staining and visualization were performed as described above.

### 2.5. Quantitative Real-Time RT-PCR

Total RNA was isolated using an RNA Purification Kit (Thermo Fisher Scientific). Total RNA (200 ng) was treated with RNase-free DNase (Sigma Aldrich) for 15 min. Following the inactivation of DNase with Ethylenediaminetetraacetic acid (EDTA) and heating, RNA was reverse transcribed using a First Strand cDNA Synthesis Kit (Thermo Fisher Scientific) according to the manufacturer’s instructions. Quantitative real time polymerase chain reaction (RT-PCR) (qPCR) was performed with cDNA samples using the Power SYBR Green Master Mix and Mic qPCR Cycler (Bio Molecular Systems). The relative mRNA level was calculated as values of 2^(Ct(β-actin) − Ct(gene of interest))^. For data presentation, the mRNA level in control cells was set to 1. The sequences of the forward and reverse primers are as follows: β actin, 5’-GGCTGTATTCCCCTCCATCG-3’ and 5’-CCAGTTGGTAACAATGCCATGT-3’; *Ccna1*, 5’-TGATGCTTGTCAAATGCTCAGC-3’ and 5’-AGGTCCTCCTGTACTGCTCAT-3’; *Ccna2*, 5’-GCCTTCACCATTCATGTGGAT-3’ and 5’-TTGCTGCGGGTAAAGAGACAG-3’.

### 2.6. Chromatin Immunoprecipitation (ChIP) Assay

Approximately ten millions of cells were crosslinked with 1% formaldehyde for 15 min, followed by quenching with 125 mM glycine for 5 min. Cells were lysed in lysis buffer containing 1% SDS, 10 mM EDTA and 50 mM Tris (pH 8.0) and then sonicated 15 times for 30 s each. The sheared DNA was diluted in dilution buffer (0.01% SDS, 1.1% Triton X-100, 1.2 mM EDTA, 16.7 mM Tris-HCl (pH 8.0) and 150 mM NaCl) and incubated with anti-rabbit IgG and anti-Fubp1 IgG overnight at 4°C. The next day, lysates were incubated with Dynabeads^®^ Protein G (Thermo Fisher Scientific) for 3 h and precipitates were eluted with 1% SDS/0.1 M NaHCO_3_. 20 μL of 5 M NaCl was added to the 500 μl eluate for reverse crosslinking and DNA was purified after treatment with 20 μg (20 μL of 1 mg/mL) of RNase A (Roche) and 40 μg (20 μL of 2 mg/mL) of Proteinase K (Roche). Enrichment was calculated based on the Ct values of qPCR and plotted as a fraction of the total input. The sequences of primers are as follows: Ccna1 Region 1, 5’-AAGGTTTTCATTAATTTTAG -3’ and 5’-GCAACATAGGCCAGCCAAAG-3’; Ccna1 Region 2, 5’-TGTCCCCTCCCAAGCCTGTC-3′ and 5′-TGCCTACGCGACTTCCTGGA-3’; Ccna1 Region 3, 5’- GGCCGCGCAGGAGACGTTGG-3’ and 5’-GGCTGACGGCGGCTTACACA-3’; Ccna1 Region 4, 5’-CGCCAGGACGATGAGGACCC-3’ and 5’-CGCCACTGAGGACTTTCGAC-3’.

### 2.7. Patients and Samples

Tumor specimens and adjacent malignant lung tissue specimens were provided by the National Biobank of Korea, Kyungpook National University Hospital (KNUH), supported by the Ministry of Health, Welfare and Family Affairs. 

### 2.8. Statistical Analysis

The unpaired two-tailed Student’s t-test was used for experiments comparing two sets of data unless noted. All results are expressed as mean ± s.e.m. GraphPad Prism software (version 6, San Diego, CA, USA) was used for all statistical analyses. Differences were considered significant when * *p* < 0.05, ** *p* < 0.01 and *** *p* < 0.001.

## 3. Results

### 3.1. Cyclin A Is a Transcriptional Target of Fubp1

Given that a main function of Fubp1 is a transcription control, we explored candidate targets of Fubp1 by analyzing two different publicly available gene expression datasets (GSE108537 and GSE4914; http://www.ncbi.nlm.nih.gov/geo/) [17,28]. These data included gene expression profiles in neural stem/progenitor cells (GSE108537) and human primary skin fibroblasts (GSE4914) after Fubp1 was silenced. To examine the role of Fubp1 in cell cycling, we focused on the expression level of genes affecting cell cycle progression. Interestingly, both datasets commonly showed that the mRNA expression levels of genes promoting cell cycling tended to be reduced under Fubp1 downregulation. In contrast, mRNA levels of genes inhibiting cell cycling examined were commonly upregulated in Fubp1-downregulated cell (Supplementary Figure S1A,B).

**Figure 1:**
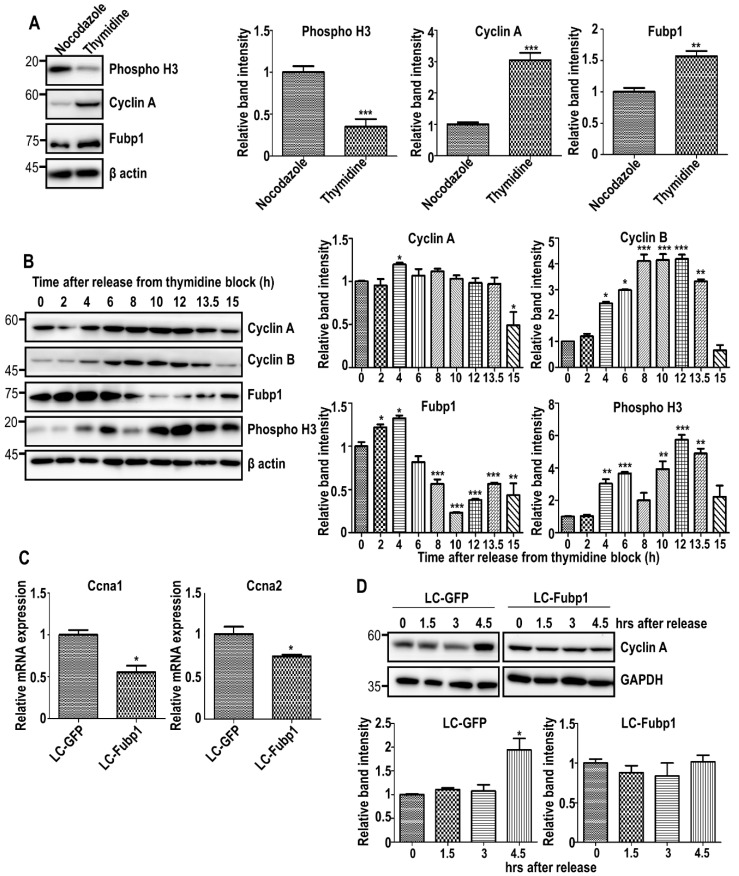
Regulation of cyclin A expression by Fubp1 during cell cycle progression. (**A**) Western blot analysis of Fubp1 after cell synchronization at the G1/S boundary via thymidine treatment and G2/M phase via nocodazole treatment. Cyclin A and phospho-Histone3 were used to confirm cell synchronization. β actin was used as a loading control. The relative band intensities of phospho-Histone 3 at Ser 10, cyclin A and Fubp1 proteins are shown in the right panels. The intensities of nocodazole-treated samples were arbitrarily set to 1. (**B**) Western blot analysis of the indicated proteins in NIH3T3 fibroblasts released from double thymidine block. β actin was used as a loading control. The relative band intensities of cyclin A, cyclin B, Fubp1 and phospho-Histone 3 at Ser 10 proteins are shown in the right panels. The intensities of samples at 0 h were arbitrarily set to 1. (**C**) mRNA levels of the *Ccna1* and *Ccna2* in LC-GFP and LC-Fubp1 cells were measured by quantitative real time polymerase chain reaction (qRT-PCR). Values are means ± s.e.m. The mRNA level in LC-GFP cells was set to 1. (**D**) Western blot analysis of cyclin A in control and Fubp1-deficient cells released from double thymidine block. GAPDH was used as a loading control. The relative band intensity of cyclin A protein is shown in the graph below. The intensities of samples at 0 h were arbitrarily set to 1. * *p* < 0.05, ** *p* < 0.01, *** *p* < 0.001.

To test the cell cycle stage-dependent role of Fubp1, we induced cell cycle arrest at the G1/S boundary and G2/M phase by treating cells with 2 mM thymidine and 100 ng/mL nocodazole for 18 h, respectively. Cell synchronization was confirmed by pH3 and cyclin A immunoblotting. Interestingly, the Fubp1 protein level was more enriched in G1/S than G2/M (Figure 1A). Due to toxicity issues when using nocodazole, we additionally performed a double thymidine block experiment. After cells were synchronized at the G1/S boundary and released into the cell cycle, the level of Fubp1 was monitored at the indicated time points. Consistently, Fubp1 protein abundance remained high in early time after release. However, it was significantly decreased while cyclin B and pH3 were accumulated, suggesting that Fubp1 is required more in the S phase than in the mitotic phase (Figure 1B).

The S-cyclins include cyclin A1 and cyclin A2, which are generally referred to as cyclin A. Among cell cycle-related genes, we focused on cyclin A as the candidate transcriptional target of Fubp1 due to following two reasons. First, transcriptome analyses demonstrated that the transcript levels of the *Ccna1* gene encoding cyclin A1 and *Ccna2* gene encoding cyclin A2 were clearly downregulated in Fubp1-silenced cells (Supplementary Figure S1A,B). Second, our data showed that the abundance of cyclin A is highly correlated with the Fubp1 level during cell cycle progression (Figure 1A,B). As we previously generated control (LC-GFP) and Fubp1-deficient (LC-Fubp1) NIH3T3 fibroblasts by utilizing the CRISPR/Cas system [29], we used these cells to experimentally test our hypothesis. As expected, both *Ccna1* and *Ccna2* transcript levels were significantly reduced in Fubp1-deficient cells (Figure 1C). Consistent with this data, the level of cyclin A protein did not properly increase in LC-Fubp1 cells following release from the double thymidine block (Figure 1D). Together, our data suggest that Fubp1 abundance is dynamically regulated during the cell cycle and that it might contribute to cell cycle progression by upregulating cyclin A expression.

Based on the previous report predicting candidate DNA sequences for Fubp1 binding [30], we analyzed the *Ccna1* promoter sequence and selected 4 different regions to assess the interaction with Fubp1 (Supplementary Figure S2A,B). Among them, regions 1 and 2 possessed two candidate sequences for Fubp1 binding. In contrast, region 3 had only a single potential site for Fubp1 association and region 4 contained no putative binding sites. Notably, as a result of chromatin immunoprecipitation (ChIP) assay, there was significant enrichment of the binding of Fubp1 in regions 1 and 2. Precipitated chromatins of these two regions were significantly decreased in LC-Fubp1 cells, confirming that Fubp1 authentically associates with these distal parts of the *Ccna1* promoter (Supplementary Figure S2C). In contrast, although we also observed significant enrichment of the binding of Fubp1 in regions 3 and 4, precipitated chromatins of these two regions were not decreased in LC-Fubp1 cells. Therefore, we concluded that Fubp1 does not interact with these proximal parts of the *Ccna1* promoter.

### 3.2. Fubp1 Deficiency Slows Down Cell Cycle Progression

**Figure 2:**
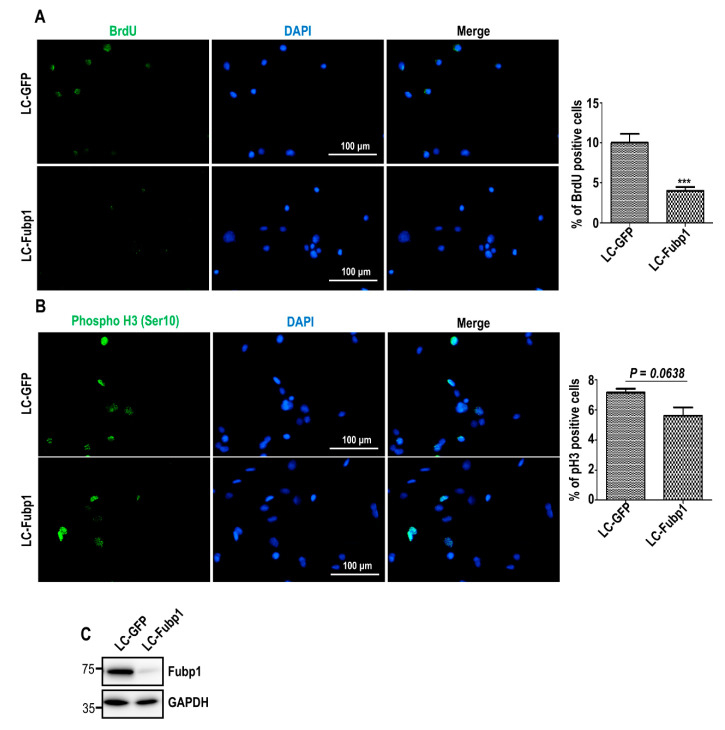
Disruption of cell cycle progression under Fubp1 deficiency. (**A**) BrdU staining was performed in LC-GFP and LC-Fubp1 cells. Green signals indicated BrdU-positive cells. Nuclear DAPI (4’,6-diamidino-2-phenylindole) staining is shown in blue. The quantification of the proportion of BrdU-positive control and Fubp1-deficient cells is shown in the right panel. *** *p* < 0.001. (**B**) Immunofluorescence analysis of phospho-Histone3 (pH3) at Ser 10 (shown in green). Nuclear DAPI staining is shown in blue. The quantification of the proportion of pH3-positive control and Fubp1-deficient cells is shown in the right panel. (**C**) Confirmation of Fubp1 deficiency by immunoblotting. GAPDH was used as a loading control. Over 800 cells were counted from 10 random fields of view per group for statistical analysis. Data were replicated in two independent experiments with similar results.

Cyclin A accumulates during the onset of the S phase and is required for DNA replication [19]. Cyclin A depletion caused the reduction of DNA synthesis activity [31]. In addition, recent evidence suggests that cyclin A could be regarded as a proliferation marker [32]. Given our data showed that Fubp1 is a transcription activator of genes encoding cyclin A, we examined the role of Fubp1 in cell proliferation. By treating control and Fubp1 deficient cells with bromodeoxyuridine (BrdU) and harvesting cells 30 min later, we detected cells undergoing DNA replication. Immunofluorescent staining for BrdU showed that the percentage of cells synthesizing DNA decreased by ~2-fold following Fubp1 deletion (Figure 2A), suggesting that Fubp1 deficiency might lead to inefficient S-phase progression. To additionally check whether Fubp1 mediates mitosis, we performed immunostaining of the mitotic marker phospho-Histone3 (pH3). As a result of counting random fields of view, the percentage of pH3-positive cells was slightly decreased with insufficient statistical significance under Fubp1 deletion (Figure 2B). Fubp1 deficiency was confirmed by immunoblotting (Figure 2C). In accordance with these results, gene profiling analysis comparing cells in states of proliferation and senescence (GEO accession: GSE100014) [33] showed enriched Fubp1 expression in proliferating cells compared with senescent cells (Supplementary Figure S1C). Furthermore, gene profiling analysis in fibroblasts after the induction of cellular senescence by heat shock factor 1 (HSF1) depletion (GEO accession: GSE111355) [34] showed that the Fubp1 transcript level was gradually decreased during senescence progression, suggesting a role of Fubp1 in cell proliferation (Supplementary Figure S1D). Together, our data suggest that Fubp1 downregulation altered cell cycle progression and the advance of S phase seemed to be more affected than that of the mitotic phase by Fubp1 absence.

### 3.3. Fubp1 Expression Patterns Show Heterogeneity among Cancer Tissues

**Figure 3:**
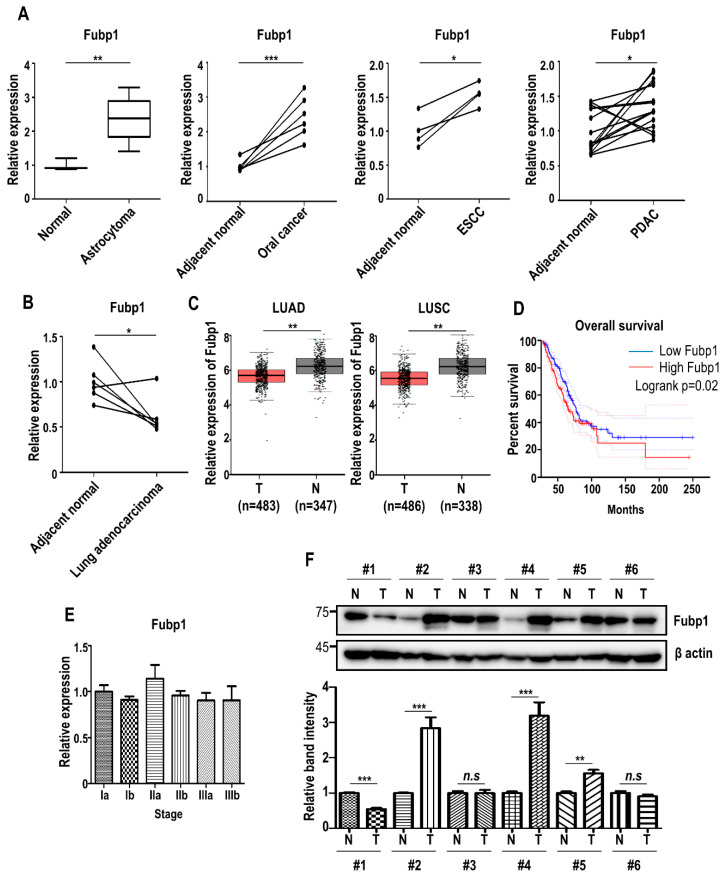
Fubp1 expression analysis in tumor tissues. (**A**) Comparison of Fubp1 mRNA expression between non-tumor and tumor cells. Transcriptome data was derived from the National Center for Biotechnology Information (NCBI) Gene Expression Omnibus (GEO) database. GEO accession: GSE15824 for astrocytoma, GSE74530 for oral cancer, GSE100942 for esophageal squamous cell carcinoma (ESCC) and GSE28735 for pancreatic ductal adenocarcinoma (PDAC). Fubp1 mRNA level was normalized with β-actin. (**B**) Comparison of Fubp1 mRNA expression between lung adenocarcinoma and adjacent normal tissue. Transcriptome data was derived from the NCBI GEO database (GEO accession: GSE118370). (**C**) Expression of Fubp1 in lung cancers evaluated by the GEPIA. (**D**) Kaplan-Meier curves of overall survival based on Fubp1 expression levels. (**E**) Comparison of Fubp1 mRNA expression during lung cancer progression. Transcriptome data was derived from the NCBI GEO database (GEO accession: GSE4573). (**F**) Western blot analysis of Fubp1 in 6 pairs of lung cancer tissues. β actin was used as a loading control. The relative band intensity of Fubp1 protein is shown in the graph below. The intensities of normal tissue samples were arbitrarily set to 1. Data were replicated in three independent experiments with similar results. N = adjacent normal, T = Tumor. * *p* < 0.05, ** *p* < 0.01, *** *p* < 0.001, n.s.—not significant.

Based on our results showing that Fubp1 has cell cycle promoting roles, we checked the mRNA expression of *FUBP1* in several types of tumors. We examined publicly available datasets containing global gene expression profiles of non-tumor and tumor tissues (GEO accession: GSE15824 for astrocytoma, GSE74530 for oral cancer, GSE100942 for esophageal squamous cell carcinoma and GSE28735 for pancreatic ductal adenocarcinoma; http://www.ncbi.nlm.nih.gov/geo/) [35,36,37,38]. Evidently, the expression of *FUBP1* was commonly upregulated in several types of cancers as previously reported (Figure 3A). However, interestingly, gene profiling analysis comparing lung cancer and adjacent normal cell (GEO accession: GSE118370) [39] revealed that *FUBP1* expression was significantly downregulated in lung adenocarcinoma, the most common lung cancer subtype among non-smokers and women (Figure 3B). In addition, we also used Gene Expression Profiling Interactive Analysis (GEPIA, http://gepia.cancer–pku.cn) [40] to look for differential gene expression and it was confirmed that the expression of *FUBP1* was clearly reduced in both lung adenocarcinoma and lung squamous cell carcinoma tissues (Figure 3C), suggesting that *FUBP1* would be less necessary in lung cancer development. In discordance with these expression patterns of *FUBP1*, however, survival analysis showed that high expression of *FUBP1* was associated with poor overall survival (OS) in lung cancer patients (Figure 3D). When we analyzed *FUBP1* expression during lung cancer progression (GEO accession: GSE4573) [41], the *FUBP1* mRNA level did not appear to be correlated with tumor stages (Figure 3E). Experimental validation of the FUBP1 protein level with immunoblot analysis in 6 pairs of lung cancer tissues showed that the FUBP1 level did not show a consistent pattern between lung cancer and adjacent normal tissues (Figure 3F). Taken together, *FUBP1* might exhibit heterogeneous and complicated functions in lung cancer development.

### 3.4. Fubp1 Is Involved in Both Cell Cycling and Cell Survival

**Figure 4:**
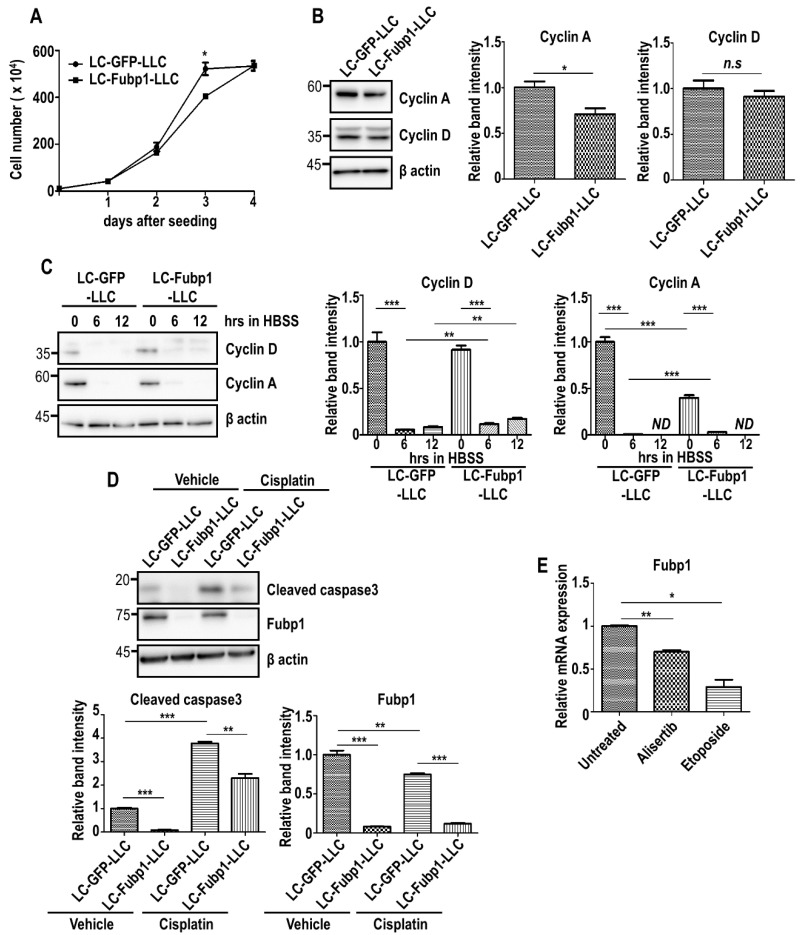
Multiple functions of Fubp1 in cell cycle progression and cell survival. (**A**) Measurement of total cell number in LC-GFP-LLC and LC-Fubp1-LLC cells. (**B**) Western blot analysis of the indicated proteins in control and Fubp1-deficient LLC cells. β actin was used as a loading control. The relative band intensities of cyclin A and cyclin D proteins are shown in the right panels. The intensities of LC-GFP-LLC cells were arbitrarily set as 1. (**C**) Western blot analysis of indicated proteins in control and Fubp1-deficient LLC cells incubated in HBSS for 6 h and 12 h. β actin was used as a loading control. The relative band intensities of cyclin A and cyclin D proteins are shown in the right panels. The intensities of LC-GFP-LLC cells at 0 h were arbitrarily set to 1. (**D**) Western blot analysis of the indicated proteins in control and Fubp1-deficient LLC cells treated with vehicle or cisplatin for 24 h. β actin was used as a loading control. The relative band intensities of cleaved caspase 3 and Fubp1 proteins are shown in the graphs below. The intensities of vehicle-treated LC-GFP-LLC cells were arbitrarily set to 1. (**E**) Comparison of Fubp1 mRNA expression in A549 lung cancer cells post treatment with anti-cancer drugs. Transcriptome data was derived from the NCBI GEO database (GEO accession: GSE102639). * *p* < 0.05, ** *p* < 0.01, *** *p* < 0.001, n.s.—not significant.

To assess the cellular mechanism of Fubp1 in lung cancer, we utilized Lewis lung carcinoma (LLC) cells. We generated control (LC-GFP-LLC) and Fubp1-deficient (LC-Fubp1-LLC) cells by using CRISPR/Cas system and monitored cell growth. Compared with control cells, Fubp1 deficiency caused a decrease in cell number at day 3 post seeding (Figure 4A). Because we previously showed that cyclin A is a transcriptional target of Fubp1 in NIH3T3 fibroblasts (Figure 1), we tested whether the Fubp1-cyclin A axis exists in LLC cells. Immunoblot analysis clearly demonstrated that cyclin A expression is reduced under Fubp1 deletion in LLC cells as well as fibroblasts, mirroring that the growth inhibition in Fubp1-deficient LLC cells is likely due to downregulation of cyclin A, at least in part (Figure 4B). In line with this data, the proportion of cells in G1 and S phases was slightly upregulated and downregulated, respectively, under Fubp1 downregulation (Supplementary Figure S4A). We additionally analyzed co-expression profiles between Fubp1 and two *Ccna* genes by utilizing cBioPortal (www.cbioportal.org) [42,43]. In other tumors we tested (bladder urothelial carcinoma, glioblastoma and head and neck squamous cell carcinoma), there was a strong and statistically significant positive correlation between the mRNA expression of Fubp1 and both *Ccna* genes, raising a possibility that this Fubp1-cyclin A axis may be ubiquitous (Supplementary Figure S3). However, Fubp1 absence did not affect the level of another cell cycle regulator, cyclin D (Figure 4B).

Interestingly, the discrepancy in cell growth between control and Fubp1-deficient LLC cells diminished by day 4 post-seeding (Figure 4A). Although this phenomenon may simply be explained by the existence of relatively more available nutrients in the culture media of LC-Fubp1-LLC cells due to a reduced cell number, it would be also possible that Fubp1 deficiency provides cells with survival advantages against metabolic stress. In order to mimic metabolic stress by nutrient deprivation in tumors, we subjected LLC cells to a prolonged incubation in Hank’s buffer salt solution (HBSS). Interestingly, LC-Fubp1-LLC cells showed higher expression of cyclin A and cyclin D compared with control cells under stress condition (Figure 4C). Consistent with this data, the proportion of Fubp1-deficient cells in the sub-G1 phase was clearly reduced (Supplementary Figure S4B,C). CCK-8 assay results also showed that cell viability of LC-Fubp1-LLC cells was higher than that of LC-GFP-LLC cells (Supplementary Figure S4D). We additionally tested whether Fubp1 deficiency provides cells with survival advantages against anti-cancer drug as well. Surprisingly, Fubp1 deletion showed a reduced level of cleaved caspase 3, indicating that lowered level of Fubp1 enhanced cell survival (Figure 4D). Gene profiling analysis in A549 lung cancer cells after treatment with anti-cancer chemotherapeutic drugs, alisertib and etoposide, (GEO accession: GSE102639) [44] showed that Fubp1 expression was significantly reduced by these anti-cancer drugs (Figure 4E). Therefore, although it seems that anti-tumor agents would be effective in the suppression of cell cycling by weakening the Fubp1-cyclin A axis, anti-tumor drugs would be inefficient in tumor extinction due to downregulation of Fubp1. Taken together, Fubp1 may play both positive and negative roles in malignant development.

## 4. Discussion

To date, many genetic studies have been performed to identify oncogenes/tumor suppressors and determine their roles in tumorigenesis and anti-tumorigenesis. However, it has been repetitively reported that the upregulation of transcripts or proteins is not necessarily caused by DNA mutation or gene amplification [25,26]. In a large diversity of neoplastic diseases, several oncogenes were shown to be overexpressed without alteration in DNA sequences or chromosome configurations [45,46]. Therefore, transcriptome expression analysis should also be solemnly performed in cancer studies. Our experimental results and supportive data driven by the analysis of publicly available gene expression datasets would be a small step toward finding out the master regulator of gene expression in several types of cancers.

The identification of novel and critical genes implicated in cancer development is an important subject for cancer research. Intriguingly, previous studies demonstrated that a group of genes exhibit both oncogenic and tumor-suppressive functions [47,48]. Generally, these “double-agent” genes are proto-oncogenes with tumor-suppressive roles and at least 83 “double-agent” genes have been documented to date. According to literature evidence, “double-agent” genes are classified into 3 categories: transcription factors, kinases and others [49]. About half of the “double-agent” genes can function as transcription regulators.

Although Fubp1 was not officially regarded as a “double-agent” gene, the debate regarding the role of Fubp1 in tumorigenesis has been around for several years. Previous reports showed that inactivating mutations of *FUBP1* were detected in 15–20% of oligodendrogliomas [16,50], suggesting a tumor suppressive role of Fubp1. However, excessive expression of *FUBP1* was documented in a variety of malignancies and *FUBP1* abundance was often inversely correlated with overall survival [51], suggesting an oncogenic role of Fubp1. In the present study, we demonstrated the tumor-promoting function of Fubp1 by showing that Fubp1 transcriptionally activates cyclin A to promote cell cycle progression. However, we also demonstrated the anti-tumorigenic role of Fubp1 by presenting that the loss of Fubp1 provides cells with survival advantages against metabolic stress and anti-cancer drugs. Based on our results, we suggest that Fubp1 may be listed as a “double-agent” gene.

Although previous studies have demonstrated an accelerated proliferation rate of *FUBP1*-overexpressing tumors, the detailed molecular mechanisms of Fubp1 in cell cycle regulation were not determined. In the present study, we identified that Fubp1 deficiency altered cell cycle progression, especially in the S phase, by downregulating the mRNA expression of *Ccna* genes. Although the Fubp1-cyclin A axis still needs to be verified in various types of tumors, our data suggest that Fubp1 has a tumor-promoting function by enhancing cell cycling. Although we showed that Fubp1 is involved in the regulation of cell survival, we could not identify the precise mechanism by which Fubp1 deficiency made cells resist cell death. One possibility is that Fubp1 might modulate cellular response to oxidative stress. Tumors often encounter metabolic stress mainly due to their rapid growth and limited nutrient supply. Metabolic stress produces an excessive amount of ROS, leading to cell death [52]. Furthermore, several anti-cancer drugs, including doxorubicin, vinblastine and paclitaxel were reported to induce intracellular oxidative stress [53]. Given that Fubp1 was reported to mediate molecular and cellular responses to oxidative stress [54], it would be necessary to determine whether Fubp1 is involved in oxidative stress generation or suppression.

## Supplementary Materials

The following are available online at https://www.mdpi.com/2073-4409/9/6/1347/s1, Figure S1. Fubp1 might be involved in cell cycle progression, Figure S2. The interaction between Fubp1 and Ccna1 promoter, Figure S3. Positive correlation between Fubp1 and Ccna1/Ccna2, Figure S4. Fubpl contributes cell cycle progression and cell survival, Figure S5. Raw western blot images.
