# Supplementary material for: Multiple Functions of Fubp1 in Cell Cycle Progression and Cell Survival
Source: Cells. 2020 May 28;9(6):1347. doi: 10.3390/cells9061347 (PMC7349734; doi:10.3390/cells9061347)
Supplement: Supplementary file 1 [file cells-09-01347-s001.pdf]

Supplementary Figure 1

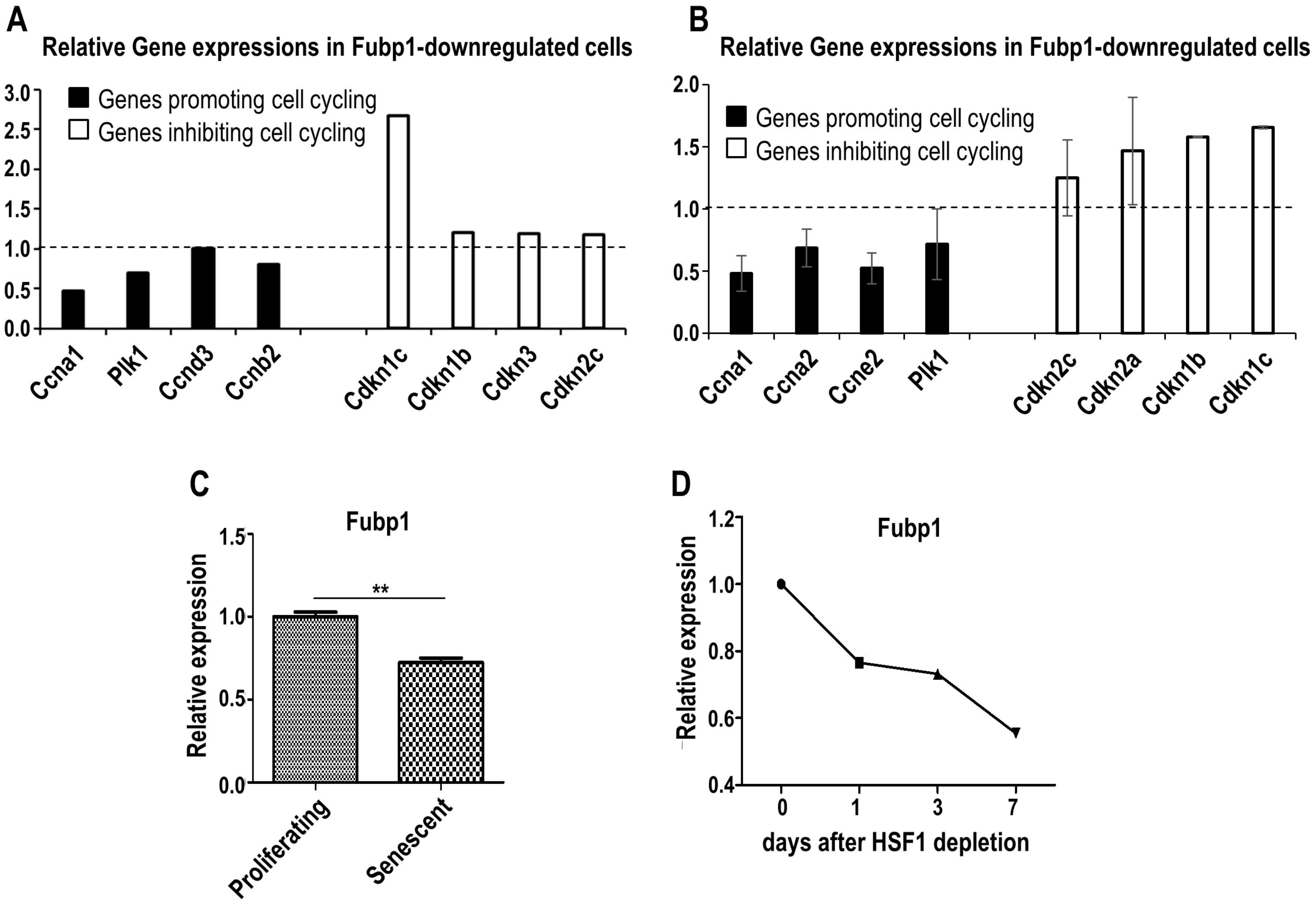

Supplementary Figure 1. Fubp1 might be involved in cell cycle progression. (A and B) mRNA expression analysis of genes promoting cell cycling (black bars) and genes inhibiting cell cycling (white bars) in Fubp1-downregulated mouse neural progenitors (A) and human primary skin fibroblasts (B). Data was extracted and analyzed from NCBI GEO profile database, GSE108537 for (A) and GSE4914 for (B). (C) Fubp1 expression data comparing normal human bronchial epithelial (NHBE) cells in states of proliferation and senescence. Data was extracted and analyzed from NCBI GEO profile database (GEO accession: GSE100014). (D) The relative transcript level of Fubp1 in human fibroblast after induction of cellular senescence by HSF1 depletion. Data was extracted and plotted from NCBI GEO profile database (GEO accession: GSE111355).

Supplementary Figure 2

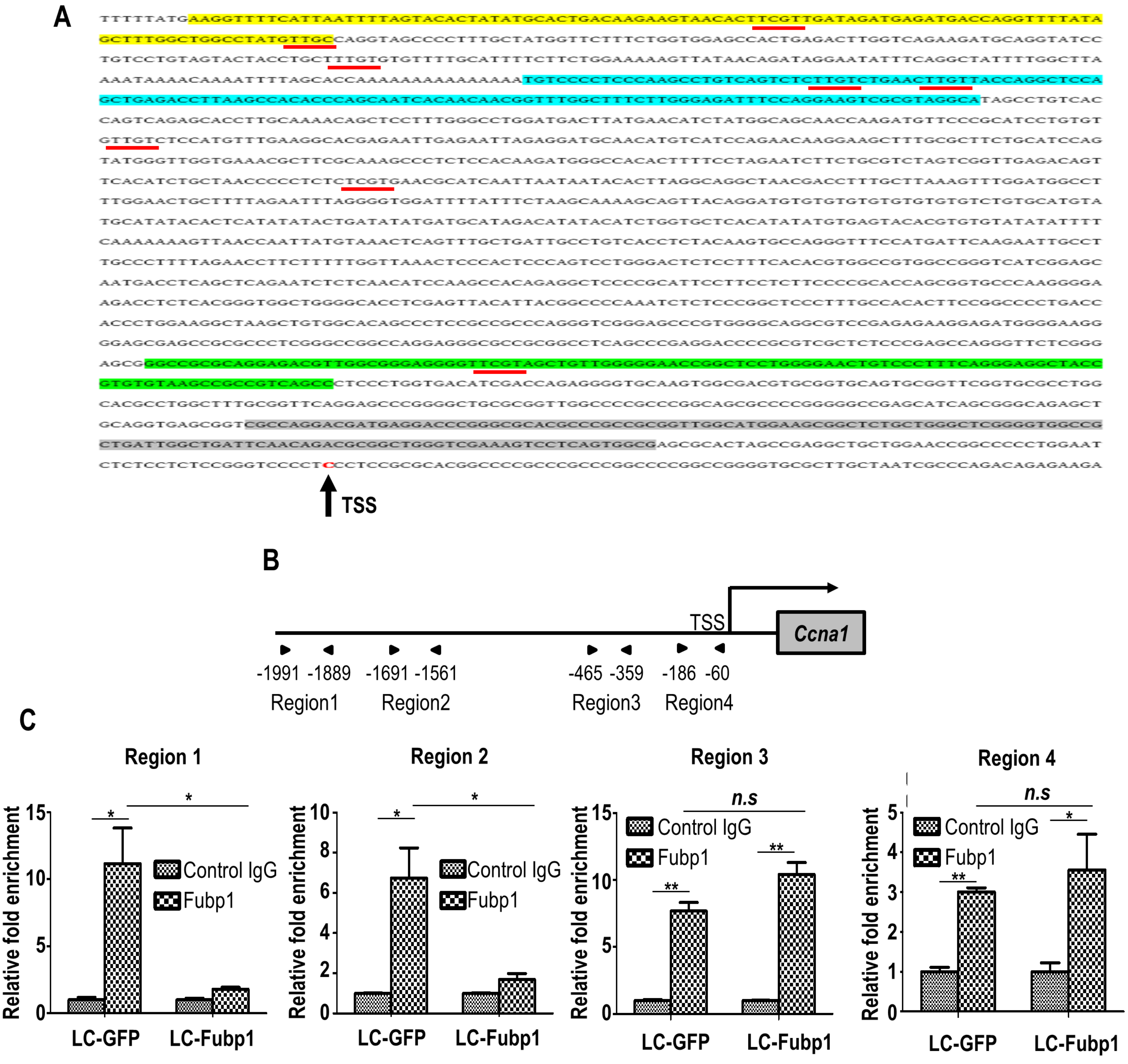

Supplementary Figure 2. The interaction between Fubp1 and Ccna1 promoter. (A) DNA sequence analysis of Ccna1 promoter. Candidate sequences for Fubp1 binding are underlined in red. Four different regions were selected for chromatin immunoprecipitation (ChIP) assay. Region 1, 2, 3, and 4 are shaded in yellow, blue, green, and grey, respectively. TSS, transcription start site. (B, C) The Fubp1 occupancy of the Ccna1 promoter was determined by ChIP. (B) Predicted Fubp1 binding regions in Ccna1 promoter. (C) qRT-PCR analysis against marked regions in the Ccna1 promoter.

Supplementary Figure 3

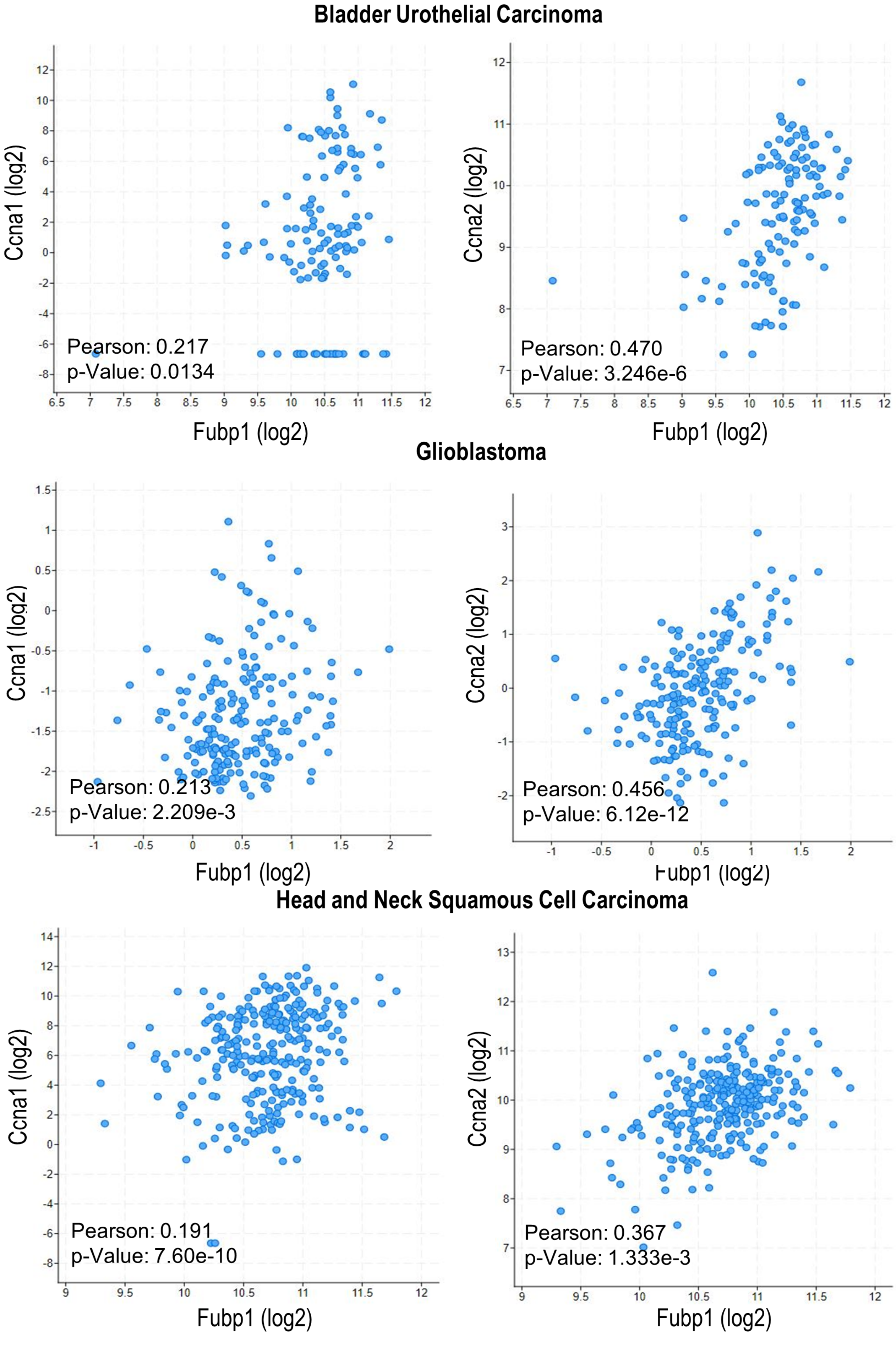

Supplementary Figure 3. Positive correlation between the expression of Fubp1 and Ccna1/Ccna2. Coexpression analysis between Fubp1 and Ccna1 (left) or Ccna2 (right) in 3 different tumor types. Data was derived from cBioPortal.

Supplementary Figure 4

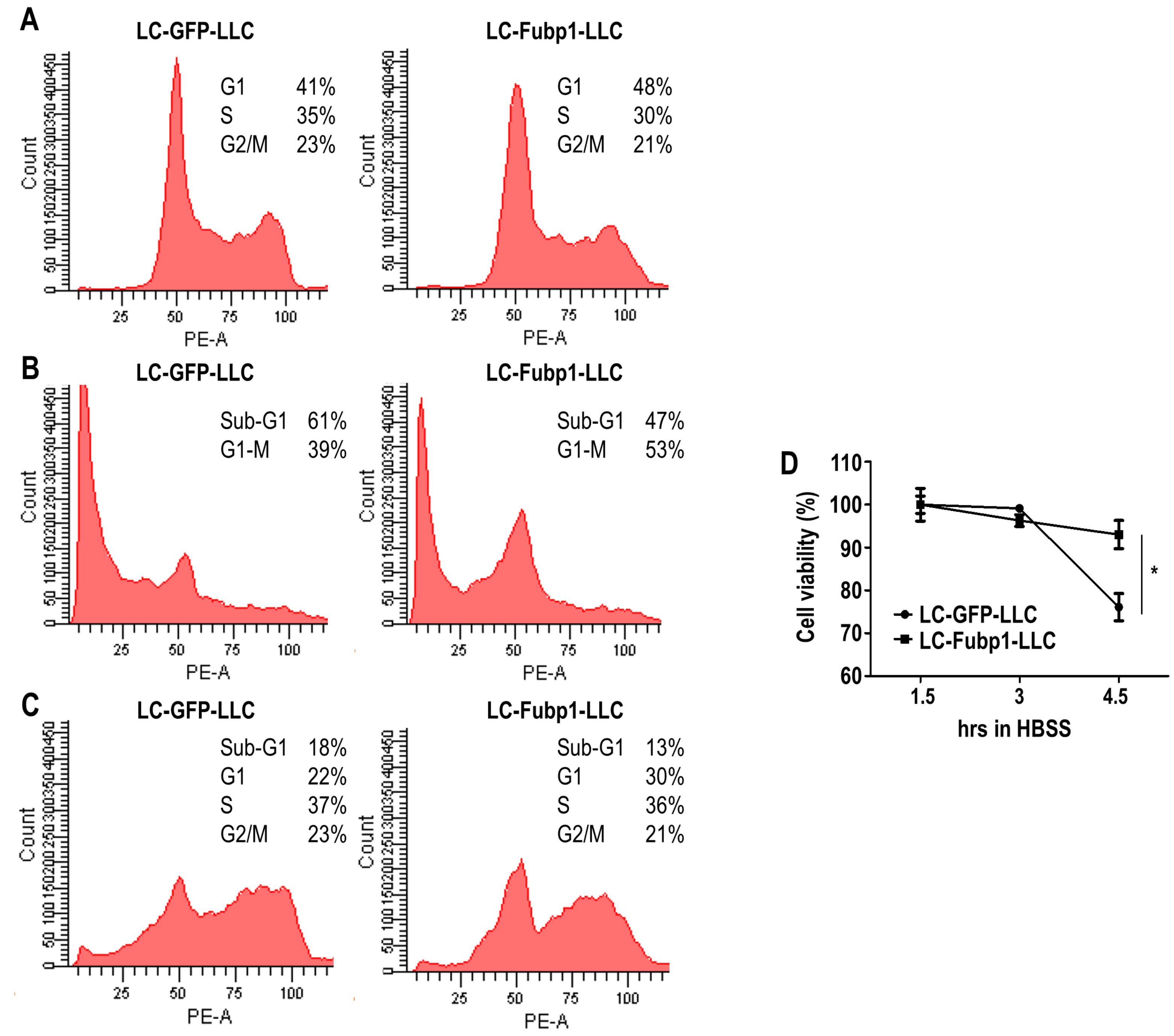

Supplementary Figure 4. Fubp1 contributes cell cycle progression and cell survival. (A) The cell cycle distributions of LLC cells were determined via flow cytometry following Fubp1 depletion. (B) The cell cycle distributions of control and Fubp1-deficient LLC cells were determined via flow cytometry after inducing metabolic stress. (C) The cell cycle distributions of control and Fubp1-deficient LLC cells were determined via flow cytometry at 4 hrs after release from the double thymidine block. (D) The results of CCK-8 assays in control and Fubp1-deficient LLC cells incubated in HBSS for 1.5 hrs, 3 hrs, and 4.5 hrs. Data are presented as the means ± SD (error bars) from three independent experiments. The cell viability of LC-GFP-LLC cells at 1.5 hrs was arbitrarily set to 1. *$P<0.05$.

Supplementary Figure 5

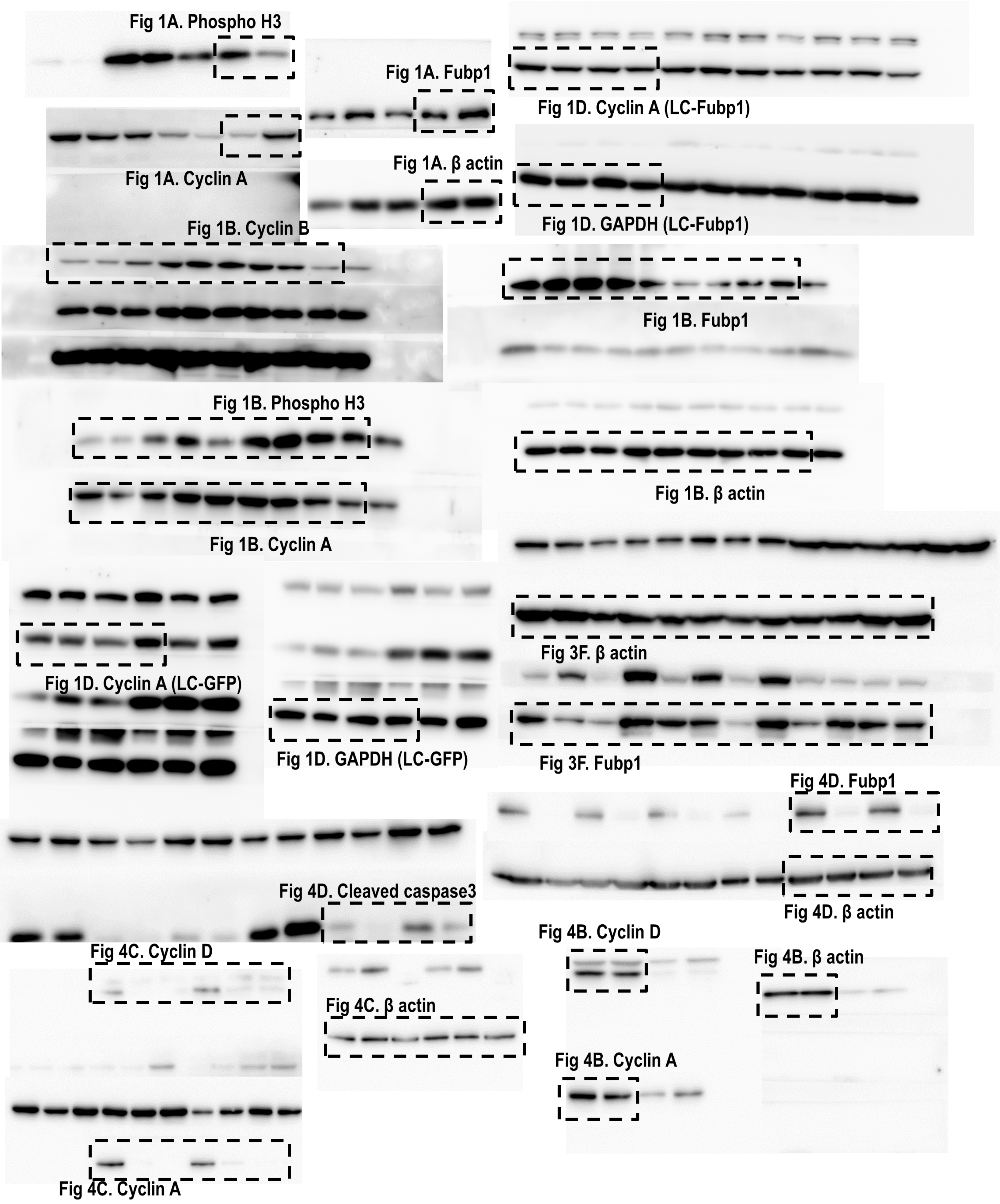

Supplementary Figure 5. Raw western blot images.
